# Correlation analysis of two-dimensional gel electrophoretic protein patterns and biological variables

**DOI:** 10.1186/1471-2105-7-198

**Published:** 2006-04-10

**Authors:** Werner Van Belle, Nina Ånensen, Ingvild Haaland, Øystein Bruserud, Kjell-Arild Høgda, Bjørn Tore Gjertsen

**Affiliations:** 1 Bioinformatics Group, Norut IT, Research Park Tromsø, Postboks 6434, N9294 Tromsø, NO, Norway; 2 lnstitute of Medicine, Hematology Section University of Bergen, Bergen, NO, Norway; 3 Earth Observation Group, Norut IT, Research Park Tromsø, Postboks 6434, N9294 Troms0, NO, Norway; 4 Department of Internal Medicine, Hematology Section Haukeland University Hospital, Bergen, NO, Norway

## Abstract

**Background:**

Two-dimensional gel electrophoresis (2DE) is a powerful technique to examine post-translational modifications of complexly modulated proteins. Currently, spot detection is a necessary step to assess relations between spots and biological variables. This often proves time consuming and difficult when working with non-perfect gels. We developed an analysis technique to measure correlation between 2DE images and biological variables on a pixel by pixel basis. After image alignment and normalization, the biological parameters and pixel values are replaced by their specific rank. These rank adjusted images and parameters are then put into a standard linear Pearson correlation and further tested for significance and variance.

**Results:**

We validated this technique on a set of simulated 2DE images, which revealed also correct working under the presence of normalization factors. This was followed by an analysis of p53 2DE immunoblots from cancer cells, known to have unique signaling networks. Since p53 is altered through these signaling networks, we expected to find correlations between the cancer type (acute lymphoblastic leukemia and acute myeloid leukemia) and the p53 profiles. A second correlation analysis revealed a more complex relation between the differentiation stage in acute myeloid leukemia and p53 protein isoforms.

**Conclusion:**

The presented analysis method measures relations between 2DE images and external variables without requiring spot detection, thereby enabling the exploration of biosignatures of complex signaling networks in biological systems.

## Background

Two-dimensional gel electrophoresis (2DE) has been a successful technique for identification and visualization of post-translational modifications [1] (reviewed in [2]), and is increasingly used to determine accessible parts of the proteome in human cells [3]. To a certain extent has 2DE been used to propose diagnosis or clinical classification in diseases [4-9], including differentiating acute myeloid leukemia (AML) from acute lymphoblastic leukemia (ALL) [10]. The amount and complexity of data obtained from 2DE patterns have led to the development of analysis software for digitalized images [11-13], but human interpretation and validation of the data is usually necessary. Typically, one of the steps in 2DE analysis is the selection of spots followed by description of their position, volume and other variables. Current methods for spot detection assume regular spot shapes [14] or model spots as bivariate Gaussian densities [15], and therefore cannot discriminate spot shapes and irregularity [16,17]. In this paper we present a method that omits the spot detection phase and does not require human interpretation on a gel-to-gel basis.

Given a set of gel images, the technique measures correlation between every pixel position and an external variable. This makes it possible to study the 2DE protein distribution as well as the actual relation to the external variable. The method has been rigorously tested on a set of simulated 2DE images with different levels of background, additional noise and outliers. Biological evaluation of the technique was performed by testing the correlation analysis on p53 protein isoform profiles in cell samples from patients with well-characterized hematological malignancies.

Different hematological malignancies, like ALL and AML [18] are characterized by distinct mutations or expression of genes involved in cell signaling [19,20]. The TP53 gene is frequently mutated in many cancers and mutations in signaling pathways acting on p53 protein are found both in sporadic and hereditary cancers [21]. The p53 protein is a sequence specific transcription factor that can regulate differentiation, growth and cell death, and is highly regulated by post-translational modifications caused by multiple signaling networks that directly or indirectly target the protein [22,23]. During differentiation, p53 undergoes modifications like phosphorylation and acetylation and is suggested to be involved in differentiation of AML [24,25]. Because of this large range of activities and complex regulatory functions, we relied on analysis of the post-translationally modified p53 protein to illustrate our method. The p53 protein biosignatures in 39 AML patients and 8 ALL patients were analyzed by 2DE immunoblot. Distinct p53 biosignatures correlated with cancer type (AML versus ALL) and, within the AML group, p53 biosignatures correlated with the level of differentiation, using the French-American-British (FAB) classification.

## Results

### Overview of the method

**Figure 1:**
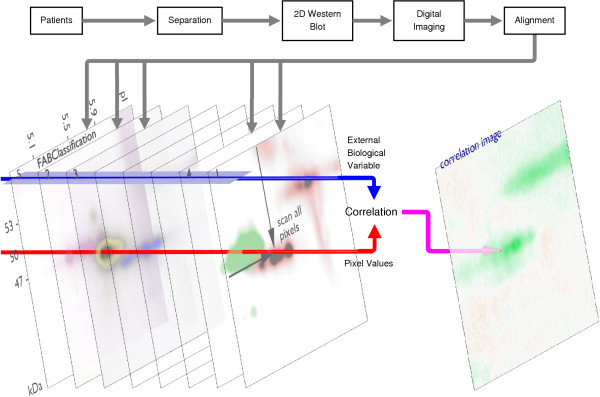
**2DE Image Correlation**. 2DE image correlation relies on an aligned, normalized stack of 2DE images and a numerical label associated with every gel. Pixel per pixel correlation between gel intensities (red arrow) and the external variable (blue arrow) creates a new image, showing areas in the gel that relate to the external parameter. In comparison to standard gel analysis methods, spot detection is not necessary and therefore less bias is introduced into the analysis process. This technique also recognizes moving spots and spot shapes that change.

The presented method relies on the basic assumption that if spots on 2DE images have biological relevance, then so must the pixels comprised within those spots. Therefore it must be possible to analyze 2DE images for correlation, without performing a spot detection step. The method requires the availability of a properly aligned stack of gel images. Each of the images must have an associated parameter *t*. Practically, *t *can represent any biological variable such as life expectancy, differentiation stage of a cell sample, age of an organism, origin of a cancer cell sample, effect of cancer therapy, cell size or even variables such as time, temperature, pressure, and so on. For every coordinate in the 2DE image stack, a correlation analysis is performed between the pixel data gathered at that position and the external variable *t*. The correlation image is then created by repeating this process at every possible position. The work-flow and the concept behind the correlation method is illustrated in Fig. 1. A movie of the method is available [see Additional file 1].

**Figure 2:**
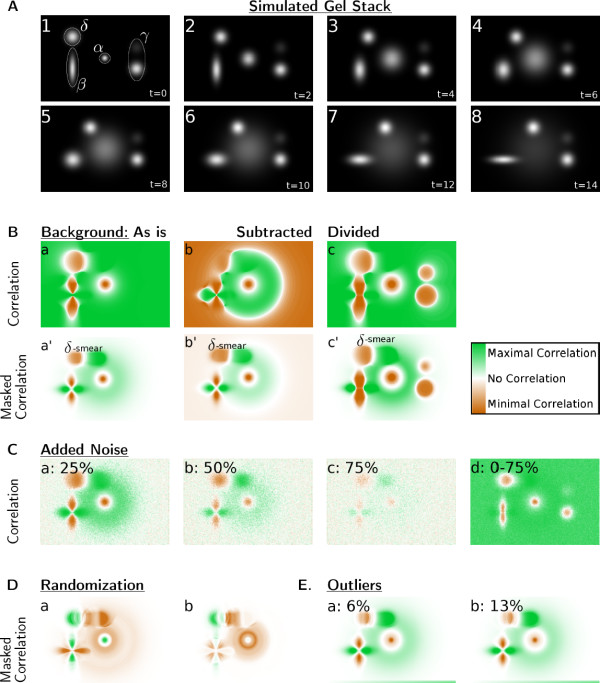
**Correlation towards a simulated 2DE gel-stack**. (A) Eight snapshots taken from a stack of 15 simulated gels generated using Gaussian bumps. Each image contains simulated spots with particular characteristics. See Material and Methods for formula and details. (B) Correlation between the gel-stack and the variable *t*. Upper gels (a-c) visualizes the correlation, lower gels (a'-c') are masked to visualizes only useful correlations. Correlation analysis was performed relying on different background removal methods. (a, a') without background removal; (b, b') with background subtraction, (c, c') using background division. (C) Correlation analysis under the presence of normal distributed noise: (a) 25%, (b) 50%, (c) 75% and (d) stepwise increasing noise from 0% to 75%. (D) Correlations towards two randomized sets of biological parameters. (E) Correlation towards the variable *t *polluted with (a) 6% outliers and (b) 13% outliers.

To illustrate how the correlation images ought to be interpreted, a simulated gel stack with defined spot characteristics in function of an external variable *t *was created (Fig. 2). This simulation reassured a controlled environment in which the algorithmic behavior was observed.

### Simulated gels

#### Altering spot position and sizes

We first verified how the method reacts to spot location, spot size and spot shifts. The simulated gel stack has various spots behaving differently. Spot *α *grows and fades out, spot *δ *shifts from left to right, spot *β *changes shape and the *γ *spots have a constant amplitude and width (Fig. 2A). Fig 2 shows various correlation images in which the strength of a correlation is presented in shades of green (for positive correlation) and brown (for negative correlation or anti-correlation). By design, spots *α *and *β *are parametrized by *t*. In the correlation images (Fig. 2B) we find them back at the same position, showing that the correlation image offers correct positional information. The two constant *γ*-spots are independent of *t*. This results in no visible correlation in Fig. 2Bab. The (*δ*-spots shifts relates to the external variable. The correlation image reveals this by showing original and destination positions that respectively correlate, then anti-correlate. This results in a smear in the correlation image (Fig. 2B).

#### Spot shape

All images in Fig. 2B show the *α*-spot to anti-correlate in the middle and to correlate at its periphery. This is consistent with the creation of the gel-stack in which the amplitude of spot a lowers from 5.0 to 1.0 while the spots broadens from 10 to 100 pixels. Because the central spot widens, higher gel numbers will have relatively more signal in the periphery. This indicates that spots where diffusion-like alteration dominate can be detected based on the difference in correlation between the inner and outer areas. Similar behavior can be observed in the shape changing *β*-spot. The initial vertical shape (low *t*-value) anti-correlates (it disappears) while the later horizontal shape (at higher *t*-values) correlates (it appears).

#### Masking the correlation image

In the simulated gel-stack, empty areas have an almost constant intensity. For those areas, the raw correlation analysis indicates a strong correlation (Fig. 2Ba) or anti-correlation (Fig. 2Bb). There are two reasons for this. First, the area can be constant, resulting in correlations that are +∞, -∞ or NaN (not a number). In the correlation image these are represented as + 1 or -1. Secondly, in areas with very small alterations (the periphery of the spots), the measured correlation is mathematically correct, but the lack in intensity variation offers little information. After applying various significance masks to the correlation image, we find that only areas with relevant spot modulations are indicated (Fig. 2(a', b', c')). One mask removes non significant correlations and a second mask removes areas without variance (see Material and Methods, Step 4 for details).

#### Effect of different normalizations

Different background removal and scaling techniques were tested on the simulated gel-stack (Fig. 2), including background subtraction and background division. In all cases, the original information that led to the creation of the gel-stack was retrieved. The *α*, *δ *and *β *spot correlations were always visualized, indicating that the normalization technique used is of little importance for qualitative analysis. In the particular case of gel normalization obtained by division through the mean gel intensity, new information was found that did not directly originate from the creation of the simulation (Fig. 2Bc). Due to a *t*-dependent intensity increase in spot *α*, the mean intensity of the gel increased. As a result, the original constant *γ*-spots decreased in intensity (division by a larger number leads to lower values). The *γ*-spots became *t *dependent and thus showed up in the correlation image.

When working with real gels this does not hinder qualitative analysis because normalization is performed on an individual gel basis. Therefore, it can always be repeated on any new gel, without taking into account previous gels and the reported correlations can be observed in the normalized images. Quantitatively, normalization factors strongly influence correlation measures. If the technique is used as a quantitative method, then calibration spots ought to be used and exact understanding of machine specifications and camera properties should be known.

#### White noise in 2DE images

In Fig. 2Ba-c, the background correlated towards *t*. Adding white noise [26] to the simulated images attenuates the appearance of such non significant background correlations (Fig. 2Ca-c. Increasing noise up to 75% (of the maximum image intensity) resulted in weaker correlations, but still important spots were identifiable (Fig. 2Cc). This suggests that small amounts of noise might enhance interpretation of the correlation analysis by automatically introducing a non-correlating variance. The signal hidden within the noise must now compete against a non-correlating factor, as such, the noise introduces a form of automatic significance measurement. When the noise amplitude is *t *dependent, we observe correct information about the negative correlation, but loss of information about the positive correlation (Fig 2Cd). Such a situation could occur if a camera automatically gates images at waning signal strength. As long as white noise does not relate to the external variable, its presence barely influences the analytical power of the presented correlation test.

#### Effect of randomization of the dataset

Two sets of random data were generated to be used as *t*-value. Instead of testing correlation towards the sequence number *t*, we now determined the effect of correlation of the images towards a random vector. The IDL function 'randomu' [27], generated the normally distributed random numbers. In the correlation images we always recognized the same general shapes. Areas that behaved similarly in the gel stack, had the same coloring, regardless of the external variable. These examples emphasize the robustness of the algorithm to group together regions of interest (Fig. 2D).

#### Outliers

A test with outliers in the *t*-values shows limited impact on the interpretation of the gels (Fig. 2E). We changed the *t*-values from {0, 1, 2, 3, 4, 5, 6, 7, 8, 9, 10, 11, 12, 13, 14} to {0, 1, **15**, 3, 4, 5, 6, 7, 8, 9, 10, 11, 12, 13, 14}, resulting in a slight change in actual correlation magnitude, but the information content was well preserved. Even with 13% outliers {0, 1, **15**, 3, 4, 5, 6, **4**, 8, 9, 10, 11, 12, 13, 14}, the original information was recovered. This is mainly due to the robust correlation which relies on ranking of the dataset instead of the numerical values (both the *t*-values and the image pixel values are ranked).

### Correlation analysis of p53 biosignatures in acute leukemia

Recently we demonstrated that signaling networks may be altered and potentiated in cancer cells suggesting a prognostic meaningful classification [28,29]. This includes altered p38 MAP-kinase signaling, known to phosphorylate p53. The application of the presented method was tested on p53 biosignatures of human primary cancer cells. The p53 biosignature is probably formed by the combinations of splice forms of p53 and various post-translational modifications [22,30]. The p53 protein is also involved in several positive and negative feedback networks [23]. This has ignited the hypothesis that p53 integrates information from various signaling networks [31].

We investigate two different relations. One illustrates a relation between the *overall *p53 intensity and AML/ALL classification, the other illustrates detection of p53-*isoform *biosignatures related to the AML FAB classification.

#### Correlation of p53 protein biosignatures towards AML/ALL

**Figure 3:**
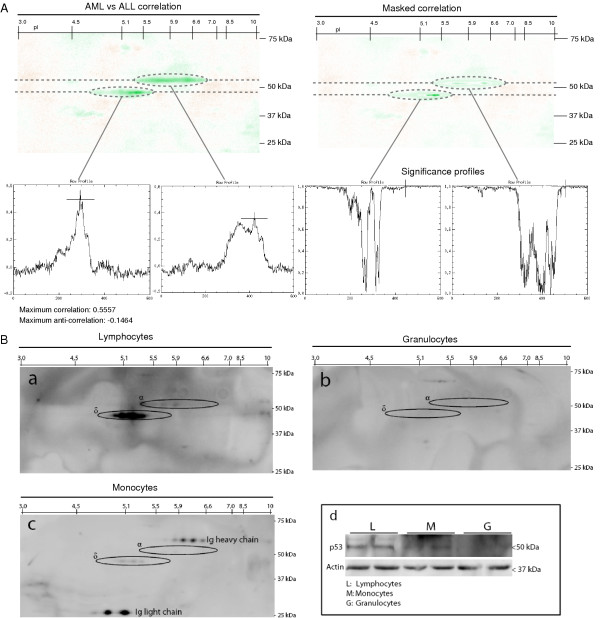
**Correlation of p53 isoforms towards cancer type (AML or ALL)**. (A) Correlation analysis of p53 2DE immunoblots from AML and ALL patient samples. A total of 73 immunoblot images from AML and 16 images from ALL were analyzed (left, correlation; right, masked correlation). Green color indicates positive correlation for ALL (maximum positive correlation 0.5557), and brown color indicates negative correlation (maximum negative correlation 0.1464). (B) p53 protein expression in mature lymphocytes, neutrophile granulocytes, and monocytes from healthy donors, examined by 2DE (a-c) and one-dimensional immunoblot (d). For comparison, protein extract from the different normal cell types were analyzed by one-dimensional gel electrophoresis and immunoblotting (d). In the monocyte isolates, immunoglobulin subunits (heavy chain, light chain) from the isolation procedure were detected in addition to weak p53-*δ *spots (c). Parallel samples of lymphocytes, monocytes and neutrophile granulocytes were analyzed by one-dimensional gel electrophoresis for direct comparison of the protein level in the different cell types on the same immunoblot (d). All immunoblots shown are representative for three or more performed experiments. See Material and Methods for details on cell separation and immunoblotting technique.

ALL and AML comprise different genetic abnormalities [32,33], and analysis of growth factor receptor expression and global gene expression has pointed out that the expression of receptor tyrosine kinases and signaling modulators are different [34,35]. Therefore, since the p53 protein is implied in various cancer related signaling networks, we expected to find distinct correlations between p53 expression and the AML/ALL variable. Gels of AML patients were marked with *t *= 0, while ALL variants were marked with *t *= 1. The correlations are shown in Fig. 3A. It reveals *overall *intensity increase of p53 in AML compared to ALL. There is no previous data from acute leukemia that supports this observation. To examine whether the 2DE p53 correlations analysis reflected actual p53 protein expression differences in the lymphoid and myeloid cell lineages, we examined normal lymphocytes, neutrophile granulocytes and monocytes by 2DE (Fig. 3Ba-c) and one-dimensional immunoblot (Fig. 3d). This confirmed the intensity-differences detected by the correlation analysis by reflecting actual attenuated p53 protein levels in lymphocytes compared to myeloid cells.

The impact of wrong ALL versus AML diagnosis was examined by random swapping ALL and AML labels in the AML/ALL versus 2DE image correlations. This results in lower correlation values as expected [see Additional file 3].

#### Correlation of p53 protein isoforms towards the AML differentiation level

The French-American-British (FAB) classification of AML is based on the morphologically determined stage of myeloid maturation and direction of maturation [36,37]. Recent reports indicate that the FAB classification, in particular the distinction between M1–2 and M4–5 in maturation level and direction of maturation, is associated with certain gene classes in unsupervised clustering of gene expression profiles [38,39]. It is previously described in several reports that p53 is involved in leukemic cell differentiation [24,25,40,41]. Phosphorylation of p53 Ser315 is necessary for differentiation in mouse embryonic stem cells [42], and p53 is able to direct differentiation in AML cell lines [25,41]. The p53-deficient HL-60 cell line has potential for both monocytic and granulocytic differentiation, and introduction of wild type p53 directs differentiation in the granulocytic direction [40]. Based on these reports we hypothesized that the p53 biosignatures should reflect the stage and direction of myeloid differentiation. Therefore, we measured correlations between the established routine morphological differentiation classification of AML (FAB) [21,22,35] and the p53 2DE biosignatures of the cancer cells.

**Figure 4:**
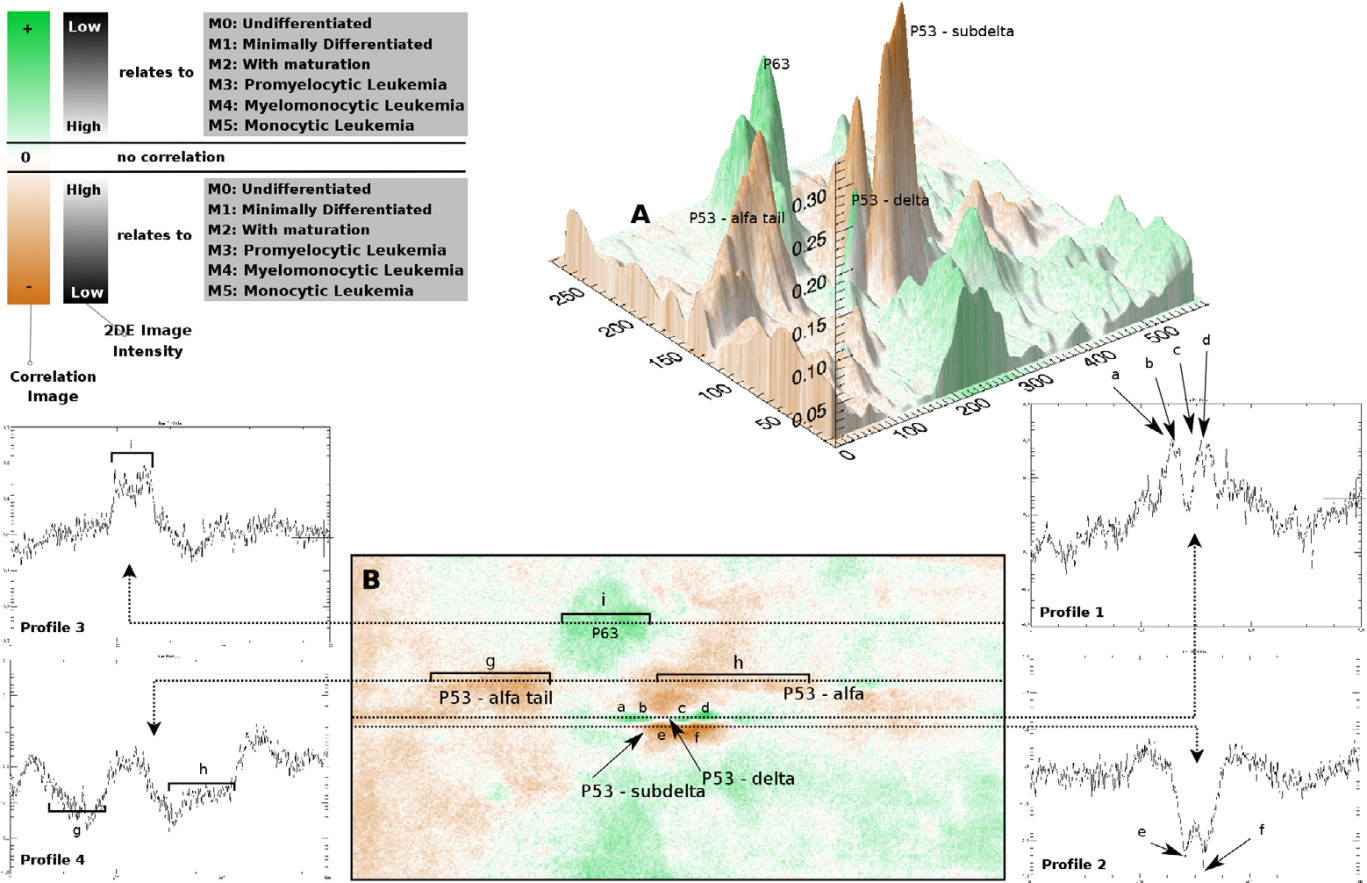
**Correlation of p53 protein isoforms towards AML differentiation (FAB)**. Green indicates correlation with more differentiated forms of AML. Such areas in 2DE images of M5 will have a higher intensity than 2DE images of M0. Brown indicates anti-correlation with the more mature forms of leukemia cells. Such areas in 2DE images of M0 will have a higher intensity than 2DE images of M5. (A) Correlation landscape of p53 in 73 AML images related to differentiation direction and stage (FAB, French-American-British classification). The vertical axis sets out the absolute correlation value. (B) Correlation image demonstrating statistical significant alterations in p53. Profile 1 shows the p53-*δ *region containing four correlating spots (r = 0.2). Profile 2 shows the sub-*δ *region anti-correlating at positions e and f. Profile 3 is the p63 region (p53 family member) correlating towards the more differentiated leukemia's. Profile 4, a p53 region anti-correlating with differentiated AML.

We assigned to every class a separate *t*-value: M0 (*t *= 0), M1 (*t *= 1), M2 (*t *= 2), M3 (*t *= 3), M4 (*t *= 4) and M5 (*t *= 5). Using 73 gels we found specific correlations (Fig. 4). Image A is the masked correlation landscape, image B is the raw correlation image. The observations were: a) The tail of the p53-*α *isoform correlates negatively to the FAB classification (profile 4, region g and h). b) The p63 area correlates positively towards the FAB classification (profile 3, the i region), c) The p53-*δ *region has four positively correlating articulated spots (profile 1, a-d, r = 0.2), d) the p53 sub-*δ *region has two negatively correlating spots (profile 2e, f). The combination of a positive correlation at the p53-*δ *region and a negative correlating sub-*δ *region indicates a spot shift from one area to another. Additionally, the e) presence of the super-*δ *negative correlating region indicates that a change of spot shape also occurs. When the p53-*δ *spots are larger and diffuse then the patient is classified as M0, M1 or M2. If the spots in the *δ *region are clear articulated and smaller, the patient is either M4 or M5. None of the above correlations are strong (*r *= 0.25 using the stringent Spearman rank order correlation). Nonetheless they can be observed in the 2DE images, which means that they can form an important tool in stratification of patients. Based on these correlation measurements, we performed further tests to verify and confirm the relation between mass-differences and the FAB classification (See section Intra-image correlations and [see Additional file 2]). The presented correlation includes M3, a distinct subgroup of AML with signs of granulocytic differentiation, featuring the translocation t(15;17) and responsiveness to retinoic acid therapy [32]. FAB M3 is therefore a separate entity in the recent WHO classification [43]. The correlations were weaker when M3 was removed (data not shown), which suggests that it is the pre-neutrophile granulocytic differentiation stage of M3 that comprises a distinct p53 isoform profile from M0/1 p53, thereby contributing to a greater splitting of the patients into subgroups.

## Discussion

### Localization, shape and volume of 2DE spots

Spot detection methods are in general very complex and time consuming tasks. The correlation technique relies on the assumption that if spots have a biological relevance then so must their individual pixels. The advantage of approaching gels this way is that we no longer depend on spot detection methods. One can wonder though, how relations between spot volumes and external variables are assessed. As it turns out, one can still rely on the correlation image because if a spot its volume changes it means that the amplitude, width (or both) have changed. As illustrated in Fig. 2, both phenomenon are detected. In general, the analysis does not favor specific shapes (such as bivariate Gaussian distributions), it will equally treat spots, tails and areas.

### Input quality

Most algorithms react differently towards different kinds of input and the quality of the result often depends on the quality of the input. Input images can have many artifacts and with 2DE the accuracy of the measurement is often unknown. We showed that our technique works surprisingly well without calibrated intensities. The use of mean background division and RMS scaling offers the same information quality as relying on exact calibrated intensities. We also observed that background noise and outliers don't influence the quality of the analysis. This is logical because we rely on ranking of the data set, therefore outliers (whether they are in the gel images or in the external variables) do not attribute any significant impact to the correlation image. This also means that *some *misaligned images will not influence the correlation image. However, when investigating alignment drift on *all *images, we find that the method quickly looses power with decreasing alignment accuracy. As the accuracy becomes less than the size of the spots, one looses analytical power [see Additional file 4]. For this method to work properly, it is thus of great importance to rely on calibration spots and use these to register the images. Especially when working with large images that can contain many thousand spots, alignment is a known problem [44,45]. Certain errors should be expected but as long as the spot jitter is smaller than the size of the spots, our algorithm will be able to provide useful results.

### Intra-image correlations

A correlation measures indicates whether two data sets relate to each other, not *how *they relate. For instance, vectors [1-3] and [2,4,6]correlate with a value 1.0 without revealing the factor 2. As such, correlation should not be confused with up- or down-regulation, nor with a causal relationship. Nevertheless, if the correlation image reveals that one area goes down in pace with the external variable while another area goes up, it is natural to ask whether the relation between these two areas is of importance. Based on the FAB/p53 correlation, we will give two examples of such intra-image relations and explain how to address them at the pixel level.

#### Does the difference between p53-*α *and p53-*δ *regions relate to the FAB classification ?

**Figure 5:**
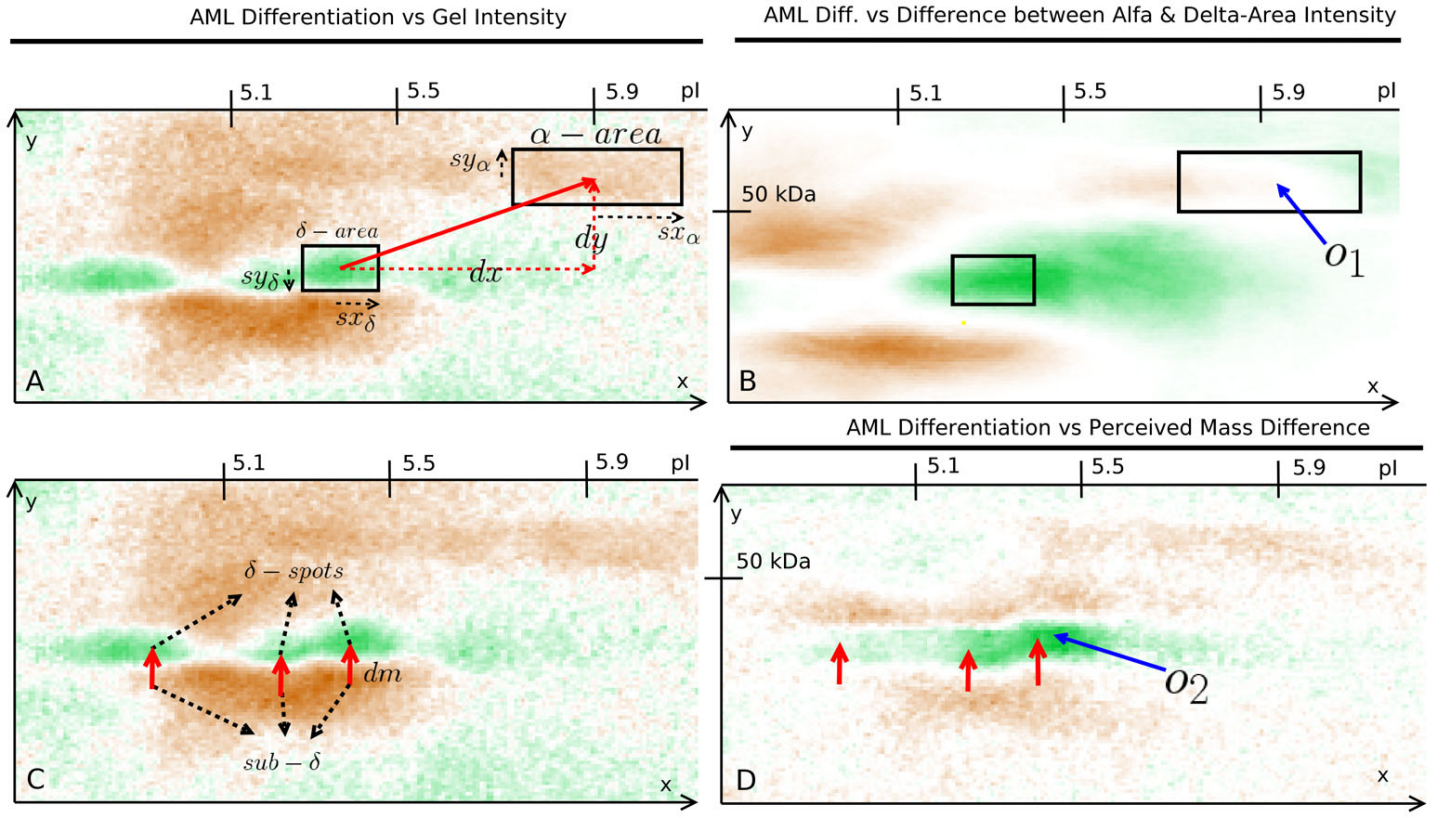
**Intra-image testing for verification whether a combination of opposing or similar correlations relates to the external variable**. When the correlation analysis reveals opposing or similar correlation in two areas, the relation between those two areas might correlate towards the external variable. Two examples are given. (A) shows that the *α*-area correlates negatively and the *δ*-area correlates positively. Does the intensity difference between the *α*-area and *δ*-area correlate with the external parameter ? To answer this, one first calculates for every image the total intensity in areas with the size of the bounding boxes of *α *and *δ*. (Their sizes are designated *sx*_*α*_, *sy*_*α*_, *sx*_*δ *_and *sy*_*δ*_). Thereafter, the images are slided over each other (the red arrow, translation *dx*, *dy*) and subtracted prior to correlation. (B) The result shows no correlation at observation point *o*_1_, indicating that the difference between *α *and *δ *does not relate to the AML differentiation stage. (C) Given the positive correlation in the *δ*-region and negative correlation in the sub-*δ *region, we want to determine whether a mass change relates to the AML differentiation stage. Image preprocessing consists of shifting the image upwards (along the red arrow, which is parallel to the mass axis) and subtracting, it from the original prior to correlation. (D) The result at observation point *o*_2 _indicates that a mass change of p53-*δ *strongly correlates to AML differentiation.

Fig. 4 reveals that the p53-*α *intensity increases with higher differentiation while the p53-*δ *intensity decreases. Therefore, we wondered whether the difference between p53-*α *and p53-*δ *areas related to the FAB classification. To answer this question, we preprocessed the images to introduce the 'difference'. This was achieved by summarizing the areas of interest and then subtracting those areas prior to correlation. Fig. 5 shows the bounding boxes of the p53-*α *and p53-*δ *areas. Their sizes, respectively (2*sx*_*α*_, 2*sy*_*α*_) and (2*sx*_*δ*_, 2*sy*_*δ*_), were used to smooth the input images (and thus measure the total intensity within such areas). The shift between the area centers (*dx*, *dy*) was used to superimpose the *δ *region over the *α *region prior to subtraction. If *I *is a 2DE image, then *I*^*α *^and *I*^*δ *^represented the two intermediate images

Ia,bα=∑x=a−sxαa+sxα∑y=b−syαb+syαIx,y
 MathType@MTEF@5@5@+=feaafiart1ev1aaatCvAUfKttLearuWrP9MDH5MBPbIqV92AaeXatLxBI9gBaebbnrfifHhDYfgasaacH8akY=wiFfYdH8Gipec8Eeeu0xXdbba9frFj0=OqFfea0dXdd9vqai=hGuQ8kuc9pgc9s8qqaq=dirpe0xb9q8qiLsFr0=vr0=vr0dc8meaabaqaciaacaGaaeqabaqabeGadaaakeaacqWGjbqsdaqhaaWcbaGaemyyaeMaeiilaWIaemOyaigabaacciGae8xSdegaaOGaeyypa0ZaaabCaeaadaaeWbqaaiabdMeajnaaBaaaleaacqWG4baEcqGGSaalcqWG5bqEaeqaaaqaaiabdMha5jabg2da9iabdkgaIjabgkHiTiabdohaZjabdMha5naaBaaameaacqWFXoqyaeqaaaWcbaGaemOyaiMaey4kaSIaem4CamNaemyEaK3aaSbaaWqaaiab=f7aHbqabaaaniabggHiLdaaleaacqWG4baEcqGH9aqpcqWGHbqycqGHsislcqWGZbWCcqWG4baEdaWgaaadbaGae8xSdegabeaaaSqaaiabdggaHjabgUcaRiabdohaZjabdIha4naaBaaameaacqWFXoqyaeqaaaqdcqGHris5aaaa@5E5A@

Ia+dx,b+dyδ=∑x=a−sxδa+sxδ∑y=b−syδb+syδIx,y
 MathType@MTEF@5@5@+=feaafiart1ev1aaatCvAUfKttLearuWrP9MDH5MBPbIqV92AaeXatLxBI9gBaebbnrfifHhDYfgasaacH8akY=wiFfYdH8Gipec8Eeeu0xXdbba9frFj0=OqFfea0dXdd9vqai=hGuQ8kuc9pgc9s8qqaq=dirpe0xb9q8qiLsFr0=vr0=vr0dc8meaabaqaciaacaGaaeqabaqabeGadaaakeaacqWGjbqsdaqhaaWcbaGaemyyaeMaey4kaSIaemizaqMaemiEaGNaeiilaWIaemOyaiMaey4kaSIaemizaqMaemyEaKhabaacciGae8hTdqgaaOGaeyypa0ZaaabCaeaadaaeWbqaaiabdMeajnaaBaaaleaacqWG4baEcqGGSaalcqWG5bqEaeqaaaqaaiabdMha5jabg2da9iabdkgaIjabgkHiTiabdohaZjabdMha5naaBaaameaacqWF0oazaeqaaaWcbaGaemOyaiMaey4kaSIaem4CamNaemyEaK3aaSbaaWqaaiab=r7aKbqabaaaniabggHiLdaaleaacqWG4baEcqGH9aqpcqWGHbqycqGHsislcqWGZbWCcqWG4baEdaWgaaadbaGae8hTdqgabeaaaSqaaiabdggaHjabgUcaRiabdohaZjabdIha4naaBaaameaacqWF0oazaeqaaaqdcqGHris5aaaa@65D2@

These two images were then subtracted to yield *O *= *I*^*α *^- *I*^*δ*^, which was subsequently put back into the gel-stack. If there was a correlation between the *α *- *δ *difference and the FAB classification then we would find it at observation point *o*_1 _in Fig. 5. We did not find a correlation, indicating that the difference between *α*-intensity and *δ*-intensity does not relate to the FAB classification.

#### Does mass-difference relate to the FAB classification ?

Fig. 4 shows the sub-*δ *region correlating negative and the *δ *region correlating positive, indicating that a mass-difference might be related to the FAB classification. Setting up this specific question is similar to the previous, but without summation of regions. The image pre-processing measures the difference between the intensity at a certain position *y *and intensities at the same position, but with a lower mass (*y *- *dm*). If *I *is an image from the gel stack, then *O *defines the new image

*O*_*x*,*y *_= *I*_*x*,*y *_- *I*_*x*,*y*-*dm*_

When using these preprocessed images into a correlation analysis, we found that observation point *o*_2 _revealed that indeed a mass-difference relates to the FAB classification. Remembering the relative weak correlation in the FAB classification (0.2 and -0.2 at the specified areas), we now find a much strong correlation: 0.507. This illustrates how the correlation images can be used to naturally explore data sets.

### Performance

The complexity of the algorithm is linear to the size of the images and the number of images. If we have *n *images of width *w *and height *h *then the calculation time will be in the order of *O*(*w*.*h*.*n*). The memory considerations are the same because all images need to be loaded in memory. E.g; 100 images of 1024 × 1024 pixels with 16 bit gray values will require around 200 Mb of internal memory. More information on complexity measurement can be found at [46,47].

## Conclusion

The presented results demonstrated that the correlation method can provide valuable information about complexly regulated proteins in biological systems. The analysis technique can be used to measure and visualize relations between 2DE images and external (biological) variables. The correlation image is calculated based on an aligned stack of 2DE images. The resulting image can be naturally interpreted and offers information that might otherwise be unavailable (such as relevant changes in spot shape). The technique is robust, general applicable to different object types (tails, spots, areas), and allows a natural amount of spot location jitter. We also investigated calibration factors and it turned out that normalization factors barely influence the analytical power of the method.

The correlation analysis of p53 biosignatures on AML and ALL cancer cells illustrated that the method can measure relations involving the overall intensity of the biosignature. The novel findings of ALL- and AML-specific p53 bioprofiles were verified on normal cells from the lymphoid and myeloid lineages. The positive correlation for full-length and *δ*- p53 in ALL was reflected by the presence of these p53 forms in lymphocytes, while these p53 forms were absent in the myeloid granulocytes. This analysis of normal cells suggest that the p53-distinction between ALL and AML is correct.

A second analysis illustrated that the correlation method differentiates between different protein isoforms. The relation between p53 biosignature and the AML FAB classification was more complex, which allowed us to explain how intra-image relations could answer specific questions. Doing so, we observed that a mass-difference in the p53 biosignature correlated strongly towards the FAB classification, suggesting that post-translational modifications of P53 relate to AML differentiation.

Future development of the method could include adjustments and corrections for hardware-parameters such as camera warping and different kinds of noise. Canonical correlations could be used to integrate information offered by similar neighboring correlation pixels [48,49]. It could also be possible to insert clustering algorithms to pseudo-color the final image or use image segmentation algorithms to classify areas automatically [50,51]. In its present form we believe the method provides a valuable tool to explore and analyze complex biosignatures and responses from signaling networks.

## Methods

### The correlation analysis

The 2DE image correlation technique relies on a large amount of 2DE images of a biological system. Every gel needs to be described by an external numerical measure. For every *n *gels (described as *A*_*z *_in which *z *is the gel image number), there are *n *external parameters, described as *T*_*z*_. Gels can further be annotated as *A*_*x*,*y*,*z *_in which (*x*, *y*) is the position on gel number *z*. *A*_*x*,*y *_is a vector containing the intensities of all gels:

*A*_*x*,*y *_= [*A*_*x*,*y*,1 _*A*_*x*,*y*,2 _... *A*_*x*,*y*,*n*_].

#### Step 1: alignment and registration

The method requires proper direction and alignment of all gels. Presence of calibration spots facilitates this process, otherwise techniques such as Hough transformation [26,52] for gel direction measurement and cross correlation [53] for multiple gel alignment can be used. Once the gels are aligned, further basic warping and registration [45] techniques are useful to account for small shifts between the different gels. The aligned images are denoted A′z
 MathType@MTEF@5@5@+=feaafiart1ev1aaatCvAUfKttLearuWrP9MDH5MBPbIqV92AaeXatLxBI9gBaebbnrfifHhDYfgasaacH8akY=wiFfYdH8Gipec8Eeeu0xXdbba9frFj0=OqFfea0dXdd9vqai=hGuQ8kuc9pgc9s8qqaq=dirpe0xb9q8qiLsFr0=vr0=vr0dc8meaabaqaciaacaGaaeqabaqabeGadaaakeaacuWGbbqqgaqbamaaBaaaleaacqWG6bGEaeqaaaaa@2F6C@.

#### Step 2: intensity normalization

The second step normalizes the intensity values of the gels to allow for inter-gel pixel comparison. Currently, little known on the relation between pixel intensities and protein concentrations. The common assumption seems to center around linear scales. However, pixel values can be relative or gamma corrected, depending on the hardware. The wide variety of possible pixel value interpretations leads us to embrace the use of relative gray values. The simulated gel stack showed that the choice of normalization technique barely influences the final correlation image.

#### Step 2a: background intensity

The background floor of a 2DE image refers to the brightness of empty gel areas. Different capture techniques produce different background floors. Background signal can be either added to all pixel values (additive background), or it can accumulate with a decaying signal (multiplicative background). As previously observed [44], most cameras introduce a mixture of additive and multiplicative backgrounds. Removal of additive noise can be done through subtracting the mean (A″z:=A′z−A′z¯
 MathType@MTEF@5@5@+=feaafiart1ev1aaatCvAUfKttLearuWrP9MDH5MBPbIqV92AaeXatLxBI9gBaebbnrfifHhDYfgasaacH8akY=wiFfYdH8Gipec8Eeeu0xXdbba9frFj0=OqFfea0dXdd9vqai=hGuQ8kuc9pgc9s8qqaq=dirpe0xb9q8qiLsFr0=vr0=vr0dc8meaabaqaciaacaGaaeqabaqabeGadaaakeaacuWGbbqqgaGbamaaBaaaleaacqWG6bGEaeqaaOGaeiOoaOJaeyypa0JafmyqaeKbauaadaWgaaWcbaGaemOEaOhabeaakiabgkHiTmaanaaabaGafmyqaeKbauaadaWgaaWcbaGaemOEaOhabeaaaaaaaa@3801@) or median value (A″z:=A′z−median(A′z)
 MathType@MTEF@5@5@+=feaafiart1ev1aaatCvAUfKttLearuWrP9MDH5MBPbIqV92AaeXatLxBI9gBaebbnrfifHhDYfgasaacH8akY=wiFfYdH8Gipec8Eeeu0xXdbba9frFj0=OqFfea0dXdd9vqai=hGuQ8kuc9pgc9s8qqaq=dirpe0xb9q8qiLsFr0=vr0=vr0dc8meaabaqaciaacaGaaeqabaqabeGadaaakeaacuWGbbqqgaGbamaaBaaaleaacqWG6bGEaeqaaOGaeiOoaOJaeyypa0JafmyqaeKbauaadaWgaaWcbaGaemOEaOhabeaakiabgkHiTiabd2gaTjabdwgaLjabdsgaKjabdMgaPjabdggaHjabd6gaUjabcIcaOiqbdgeabzaafaWaaSbaaSqaaiabdQha6bqabaGccqGGPaqkaaa@41BE@). Removal of multiplicative noise can be done through A″z:=A′zA′z¯−1
 MathType@MTEF@5@5@+=feaafiart1ev1aaatCvAUfKttLearuWrP9MDH5MBPbIqV92AaeXatLxBI9gBaebbnrfifHhDYfgasaacH8akY=wiFfYdH8Gipec8Eeeu0xXdbba9frFj0=OqFfea0dXdd9vqai=hGuQ8kuc9pgc9s8qqaq=dirpe0xb9q8qiLsFr0=vr0=vr0dc8meaabaqaciaacaGaaeqabaqabeGadaaakeaacuWGbbqqgaGbamaaBaaaleaacqWG6bGEaeqaaOGaeiOoaOJaeyypa0ZaaSaaaeaacuWGbbqqgaqbamaaBaaaleaacqWG6bGEaeqaaaGcbaWaa0aaaeaacuWGbbqqgaqbamaaBaaaleaacqWG6bGEaeqaaaaaaaGccqGHsislcqaIXaqmaaa@390B@. We would emphasize that whatever normalization scheme is used in this step, it should be performed on an individual gel basis.

#### Step 2b: scaling of gel intensity

After removal of the background floor, the dynamic range of the image is normalized through scaling of gel intensities. The presence of a calibration spot eases this process. If *A' *is the non-relative image and (*x*, *y*) is the calibration spot position, then the image A″:=A′A′x,y
 MathType@MTEF@5@5@+=feaafiart1ev1aaatCvAUfKttLearuWrP9MDH5MBPbIqV92AaeXatLxBI9gBaebbnrfifHhDYfgasaacH8akY=wiFfYdH8Gipec8Eeeu0xXdbba9frFj0=OqFfea0dXdd9vqai=hGuQ8kuc9pgc9s8qqaq=dirpe0xb9q8qiLsFr0=vr0=vr0dc8meaabaqaciaacaGaaeqabaqabeGadaaakeaacuWGbbqqgaGbaiabcQda6iabg2da9maalaaabaGafmyqaeKbauaaaeaacuWGbbqqgaqbamaaBaaaleaacqWG4baEcqGGSaalcqWG5bqEaeqaaaaaaaa@3604@ defines the normalized image. Without calibration spot the total energy content (sum of all intensities or RMS value) forms a very reasonable scaling means: A″z=A′zRMS(A′z)
 MathType@MTEF@5@5@+=feaafiart1ev1aaatCvAUfKttLearuWrP9MDH5MBPbIqV92AaeXatLxBI9gBaebbnrfifHhDYfgasaacH8akY=wiFfYdH8Gipec8Eeeu0xXdbba9frFj0=OqFfea0dXdd9vqai=hGuQ8kuc9pgc9s8qqaq=dirpe0xb9q8qiLsFr0=vr0=vr0dc8meaabaqaciaacaGaaeqabaqabeGadaaakeaacuWGbbqqgaGbamaaBaaaleaacqWG6bGEaeqaaOGaeyypa0ZaaSaaaeaacuWGbbqqgaqbamaaBaaaleaacqWG6bGEaeqaaaGcbaGaemOuaiLaemyta0Kaem4uamLaeiikaGIafmyqaeKbauaadaWgaaWcbaGaemOEaOhabeaakiabcMcaPaaaaaa@3B52@

#### Step 3: correlation image

After alignment and normalization, the correlation analysis generates a new image visualizing the correlation measure between a specific position and an external parameter. The correlation image is composed of pixels, each testing one position on the gel. The result of each test is a number between -1.0 (anti-correlation) and 1.0 (correlation), which, after appropriate scaling, defines the pixel color in the correlation image. The two vectors participating in the test are A″x,y
 MathType@MTEF@5@5@+=feaafiart1ev1aaatCvAUfKttLearuWrP9MDH5MBPbIqV92AaeXatLxBI9gBaebbnrfifHhDYfgasaacH8akY=wiFfYdH8Gipec8Eeeu0xXdbba9frFj0=OqFfea0dXdd9vqai=hGuQ8kuc9pgc9s8qqaq=dirpe0xb9q8qiLsFr0=vr0=vr0dc8meaabaqaciaacaGaaeqabaqabeGadaaakeaacuWGbbqqgaGbamaaBaaaleaacqWG4baEcqGGSaalcqWG5bqEaeqaaaaa@31C4@ and *B*. The first vector contains the gel expression levels at position (*x*, *y*). Given 89 gel images, A″x,y
 MathType@MTEF@5@5@+=feaafiart1ev1aaatCvAUfKttLearuWrP9MDH5MBPbIqV92AaeXatLxBI9gBaebbnrfifHhDYfgasaacH8akY=wiFfYdH8Gipec8Eeeu0xXdbba9frFj0=OqFfea0dXdd9vqai=hGuQ8kuc9pgc9s8qqaq=dirpe0xb9q8qiLsFr0=vr0=vr0dc8meaabaqaciaacaGaaeqabaqabeGadaaakeaacuWGbbqqgaGbamaaBaaaleaacqWG4baEcqGGSaalcqWG5bqEaeqaaaaa@31C4@ will contain 89 different expression values; one for each gel. The second vector *B *contains 89 external values associated with every gel. Repeating this correlation test for every pixel results in the correlation image *C *(Eq. 1)

*C*_*x*,*y *_= *ρ*(A″x,y
 MathType@MTEF@5@5@+=feaafiart1ev1aaatCvAUfKttLearuWrP9MDH5MBPbIqV92AaeXatLxBI9gBaebbnrfifHhDYfgasaacH8akY=wiFfYdH8Gipec8Eeeu0xXdbba9frFj0=OqFfea0dXdd9vqai=hGuQ8kuc9pgc9s8qqaq=dirpe0xb9q8qiLsFr0=vr0=vr0dc8meaabaqaciaacaGaaeqabaqabeGadaaakeaacuWGbbqqgaGbamaaBaaaleaacqWG4baEcqGGSaalcqWG5bqEaeqaaaaa@31C4@, *T*)     (1)

The correlation image can be visualized using different color schemes. In Fig. 1 green indicates positive correlations and brown negative correlations.

The preferred correlation is the robust Spearman rank order correlation (*ρ*-correlation) [27]. This non-parametric test allows us to ignore the specific distributions of gel intensity levels and external parameters, *ρ*-correlation requires a ranking of the two participating vectors and then relies on a standard linear Pearson correlation. The ranking process will replace every value in the input vector by its specific rank. When ties occur (the same value occurring more than once) their rank will by convention be the mean of their ranks as if they all would have had a slightly different value.

#### Step 4: masking

Correlation does not necessarily imply a causal, significant, or useful relationship. To filter out some possibly useless relations, a number of masks limit the visible correlations. The first mask removes correlations that might be occurring by coincidence: some data sets easily correlate with any other data set (significance). The second mask removes correlations that offer little useful information (E.g: a data set containing all zero's).

#### Step 4a: significance

To remove correlations that have a high probability of occurring, the significance test typically associated with the Spearman correlation test was used. In this context, it is defined as

Sx,y=1−Cx,yn−21−Cx,y2     (2)
 MathType@MTEF@5@5@+=feaafiart1ev1aaatCvAUfKttLearuWrP9MDH5MBPbIqV92AaeXatLxBI9gBaebbnrfifHhDYfgasaacH8akY=wiFfYdH8Gipec8Eeeu0xXdbba9frFj0=OqFfea0dXdd9vqai=hGuQ8kuc9pgc9s8qqaq=dirpe0xb9q8qiLsFr0=vr0=vr0dc8meaabaqaciaacaGaaeqabaqabeGadaaakeaacqWGtbWudaWgaaWcbaGaemiEaGNaeiilaWIaemyEaKhabeaakiabg2da9iabigdaXiabgkHiTiabdoeadnaaBaaaleaacqWG4baEcqGGSaalcqWG5bqEaeqaaOWaaOaaaeaadaWcaaqaaiabd6gaUjabgkHiTiabikdaYaqaaiabigdaXiabgkHiTiabdoeadnaaDaaaleaacqWG4baEcqGGSaalcqWG5bqEaeaacqaIYaGmaaaaaaqabaGccaWLjaGaaCzcamaabmaabaGaeGOmaidacaGLOaGaayzkaaaaaa@48ED@

If this number is close to 1 then there exists a low probability that some random data would happen to correlate with the given result set. Likewise, if this number is 0 then there exists a high probability that the correlation is coincidental.

#### Step 4b: variance

The second mask avoids strong and significant correlations that have a low biological significance because the gel intensities do not change enough. It relies on the standard deviation [54] measured on the relative, non-ranked, gel intensities

Dx,y=∑z=0n−1(A″x,y,zA″x,y,*¯−1)2N     (3)
 MathType@MTEF@5@5@+=feaafiart1ev1aaatCvAUfKttLearuWrP9MDH5MBPbIqV92AaeXatLxBI9gBaebbnrfifHhDYfgasaacH8akY=wiFfYdH8Gipec8Eeeu0xXdbba9frFj0=OqFfea0dXdd9vqai=hGuQ8kuc9pgc9s8qqaq=dirpe0xb9q8qiLsFr0=vr0=vr0dc8meaabaqaciaacaGaaeqabaqabeGadaaakeaacqWGebardaWgaaWcbaGaemiEaGNaeiilaWIaemyEaKhabeaakiabg2da9maalaaabaWaaOaaaeaadaaeWaqaaiabcIcaOmaalaaabaGafmyqaeKbayaadaWgaaWcbaGaemiEaGNaeiilaWIaemyEaKNaeiilaWIaemOEaOhabeaaaOqaamaanaaabaGafmyqaeKbayaadaWgaaWcbaGaemiEaGNaeiilaWIaemyEaKNaeiilaWIaeiOkaOcabeaaaaaaaaqaaiabdQha6jabg2da9iabicdaWaqaaiabd6gaUjabgkHiTiabigdaXaqdcqGHris5aOGaeyOeI0IaeGymaeJaeiykaKYaaWbaaSqabeaacqaIYaGmaaaabeaaaOqaaiabd6eaobaacaWLjaGaaCzcamaabmaabaGaeG4mamdacaGLOaGaayzkaaaaaa@53B3@

The standard variance (or RMS) of the mean divided gels will have a large value where there is a varying gel expression. At places where the gel expression is constant this value will be zero.

#### Step 4c: the masked correlation image

Multiplying the standard deviation mask (Eq. 3) with the significance mask (Eq. 2) gives a new mask that can be superimposed over the correlation image (Eq. 1).

*R *= *C *× *S *× *D*

The pixel values of *R *no longer relates to the correct correlation measure. Therefore, *R *forms an indicator, showing position of possible interest.

### Simulation of a 2DE image stack

The simulated gel-stack is based on the animation of different 2D Gaussian 'bumps', defined as

G(x,y)=a.exp(−(x−cxwx)2+(y−cywy)22)
 MathType@MTEF@5@5@+=feaafiart1ev1aaatCvAUfKttLearuWrP9MDH5MBPbIqV92AaeXatLxBI9gBaebbnrfifHhDYfgasaacH8akY=wiFfYdH8Gipec8Eeeu0xXdbba9frFj0=OqFfea0dXdd9vqai=hGuQ8kuc9pgc9s8qqaq=dirpe0xb9q8qiLsFr0=vr0=vr0dc8meaabaqaciaacaGaaeqabaqabeGadaaakeaacqWGhbWrcqGGOaakcqWG4baEcqGGSaalcqWG5bqEcqGGPaqkcqGH9aqpcqWGHbqycqGGUaGlieGacqWFLbqzcqWF4baEcqWFWbaCcqGGOaakcqGHsisldaWcaaqaamaabmaabaWaaSaaaeaacqWG4baEcqGHsislcqWGJbWycqWG4baEaeaacqWG3bWDcqWG4baEaaaacaGLOaGaayzkaaWaaWbaaSqabeaacqaIYaGmaaGccqGHRaWkdaqadaqaamaalaaabaGaemyEaKNaeyOeI0Iaem4yamMaemyEaKhabaGaem4DaCNaemyEaKhaaaGaayjkaiaawMcaamaaCaaaleqabaGaeGOmaidaaaGcbaGaeGOmaidaaiabcMcaPaaa@54FB@

(*cx*, *cy*) is the center position, *wx *and *wy *are the width and height respectively, *a *is the amplitude of the curve. Based on this Gaussian 'bump' a gel-stack, containing 15 different gels was constructed. Every gel contains: I) an out-fading spot (Fig. 2, spot a) with a growing radius from 10 to 100 pixels and lowering amplitude from 5.0 to 1.0. II) An elliptical spot (Fig. 2, spot *β*) which changes shape from being small and tall (*wx *= 10, *wy *= 40, *a *= 5) to broad and flat (*wx *= 40, *wy *= 10, *a *= 5). III) Two spots with minimal (1.0) and maximal (5.0) amplitudes (Fig. 2, spots *γ*). IV) A moving spot (Fig. 2, spot *δ*) from left to right.

### Patients

The study was approved by the local Ethics Committee and samples collected after informed consent. A total of 39 unique AML and 8 ALL patients were analyzed by 2DE and immunoblotting for visualization of the p53 protein pattern by an amino-terminal targeting antibody Bp53–12. Patients were immunophenotypically classified as positive when at least 20% of the AML cells expressed the membrane molecule [55]. ALL and AML was distinguished by immunophenotyping, see Table 1 with characteristics. AML FAB differentiation classification was determined by morphological examination (microscopy) after May-Grunewald-Giemsa (MGG) staining [36,37], a cytochemical stain that predominantly reflects protein-features of the leukemia cells. Cytogenetic abnormalities in AML cells were classified according to Wheatley et al. [56]. The FAB classification is recently shown to be reflected in the gene expression of the AML cells [38,39]. The AML patients represent a consecutive group with high leukemia cell counts in peripheral blood (median blast count 67. 10^9^/L, range 17–285), and at least 80% of the peripheral blood leukocytes were AML cells. The ALL patients also represent a group of consecutive patients with high blood blast counts (median 83. 10^9^/L, range 49–560).

### Leukemic cell separation and sample preparation

Cell separation, storage and culture of patient AML blasts were performed as previously described [28,57]. ALL and AML blasts were isolated by density gradient separation with Lymphoprep (Nycomed Pharma AS, Oslo, Norway) and contained more than 95% malignant cells. Normal granulocytes (97% neutrophile) and lymphocytes (peripheral blood mononuclear cells containing 10% monocytes and predominantly T lymphocytes) were separated by density gradient centrifuging combining Polymorphprep TM (Axis-Shield PoC AS, Oslo, Norway) and Lymphoprep following the manufacturers instructions. To avoid contamination of the myeloid monocytes in the lymphocytes, lymphocytes and monocytes were separated using an autoMACS magnetic sorter (Miltenyi Biotec GmbH). CD14+ cells (monocytes) were magnetically labelled with CD 14 Microbeads (Miltenyi Biotec), following the procedure described by the manufacturer. CD14-PE antibody (Miltenyi Biotec) was used for flow cytometric determination of the purity of the two fractions (99% pure lymphocytes in flow through, 94% pure monocytes in magnetic eluate). Preparation for 2DE and immunoblotting was performed as previously described [5,58,59]. Briefly, cells were washed in NaCl (9 mg/ml) and then lysed in 7% trichloroacetic acid. The precipitated protein was washed once in 5% trichloroacetic acid and three times in water saturated ether to remove salts. The protein pellet was resuspended in sample buffer for 2DE gel electrophoresis (7 M urea, 2 M thiourea, 100 mM dithiotreitol, 1.5% Ampholyte 3 – 10, 0.5% Ampholyte 5 – 6, 0.5% CHAPS). 2D was performed using 7 cm pH 3–10 (Zoom Strip, Invitrogen Corp., Carlsbad, CA, USA) isoelectric focusing gel strips, following the manufacturers' instructions. Electrophoresis was performed at 200 V for 60 minutes, after which the proteins were transferred to polyvinylidene fluoride membrane (Amersham Biosciences AB, Uppsala, Sweden) by standard electro-blotting. p53 protein was detected using primary Bp53-12 antibody (Santa Cruz Biotechnology, CA, USA) and secondary horse radish peroxidase conjugated mouse antibody (Jackson ImmunoResearch, West Grove, PA, USA) visualized using the Supersignal West Pico or Femto Chemiluminescent Substrate system (Pierce Biotechnology, Inc., Rockford, IL, USA). Chemiluminescence imaging was performed using a Kodak Image Station 2000R (Eastman Kodak Company, Lake Avenue, Rochester, NY, USA) and were saved in TIFF format with the resolution of 300 DPI for correlation analysis.

## Availability and requirements

Project name: 2DE Correlation Analysis

Project home page: 

Operating system(s): Platform independent

Programming language: IDLv6.1 [60]

License: All licensing inquiries should be directed towards TTO Nord AS (Forskningsparken, 9294 Tromsø, Norway. Phone: +47 776 29418). The method can be freely used in academic environments. The source material [see Additional file 5] includes correlation analysis, image coloring, Gaussian bumps and the simulated images. A user friendly version of the software is being developed at .

## Abbreviations

*A *Capital letters are used to denote image matrices. An image is an element of ℝ^*w *× *h*^. *w *and *h *are the width and height of the image.

*A*_*x*,*y *_Pixel positions are written using subscript. *A*_*x*,*y *_refers to the gray value of the pixel at position (*x*, *y*) in image *A*. Subscripts are members of ℕ.

ALL acute lymphoblastic leukemia

AML acute myeloid leukemia

2DE two-dimensional gel electrophoresis

FAB The standardized French-American-British AML differentiation classification.

## Authors' contributions

WVB invented and designed the correlation algorithm, processed the digitized images, designed and performed correlation analysis simulations, and drafted the manuscript. NÅ carried out the 2DE analysis of AML and ALL patient material, aligned the 2DE images and helped drafting the manuscript. IH designed and carried out PBMC analysis and helped to draft the manuscript. ØB collected the AML biobank material, applied for ethical permission, collected clinical information and helped to draft the manuscript. BTG presented the original analysis challenge, helped to collect clinical data, coordinated the work and drafted the manuscript. All authors read and approved the final manuscript. WVB, NÅ and BTG contributed in the design of the study. KAH helped drafting the manuscript and contributed the idea to investigate the alignment accuracy.

## Supplementary Material

Additional file 1 **Animated Correlation Method**. A movie illustrating the correlation method made with blender [61]. The movie can be played with mplayer [62]. Click here for file

Additional file 3 **Correlation images illustrating impact of misclassification**. The impact of wrong ALL versus AML diagnosis was examined by random swapping ALL and AML labels in the AML/ALL versus 2DE image correlations. This results in lower correlation values as expected. Click here for file

Additional file 2 **Spot shapes**. Two images showing the difference in spot sizes between M0/M1/M2 and M4/M5 samples. The process of the changing spot distribution can be visualized by sorting all images according to their FAB classification and then showing them chronologically. This is visualized in a small movie. The two images and the movie are contained within a zip file. It can be extracted using unzip [63,64]. The movie can be played with mplayer [62]. Click here for file

Additional file 4 **Alignment accuracy**. The correlation algorithm relies on the correct alignment of input images. This process is typically performed using calibration marks on gels. The additional data illustrates the importance of correct alignment. Click here for file

Additional file 5 **Source material**. The source material includes correlation analysis, image coloring, Gaussian bumps and the simulated images. The source is contained within a .html file. The algorithm is implemented in IDLv6.1 [60]. Click here for file

## Figures and Tables

**Table 1:** Clinical and biological characteristics of acute leukemia patients.

	AML patients	ALL patients
**Number/age/gender**		
Number	39	8
Age; median (variation range)	61 (29–84)	41 (22–79)
Male/female	21/18	6/2
**Secondary leukemia**		
Previous chemotherapy	5/39	1/8
Primary MDS	5/39	
Chronic myeloid leukemia	1/39	

**Morphological classification **(FAB classification)	M0 1/39	L1 5/8
	M1 7/39	L2 3/8
	M2 11/39	
	M3 2/39	
	M4 9/39	
	M5 9/39	

**Membrane molecule analysis**	Membrane molecule expression ^1^:	Membrane molecule phenotype: T-ALL 2/8
	CD13 31/39	ALL-B lymphocyte type 6/8
	CD 14 5/39	Subclassification ^1^
	CD15 14/39	- Pro-B 2
	CD33 37/39	- Pre-pre-B 2
	CD34 17/39 (stem cell marker)	- B-ALL 2

**Genetic abnormalities**		
Number of patients examined	36	Coclusive cytogenetic analysis 3/8 Molecular analysis of bcr-abl translocation, 3 additional patients
Cytogenetic analysis ^2^	Normal 15/36	Normal karyotype 0
	Intermediate 13/36	Multiple abnormalities 1
	Favourable 3/36	Single, high-risk abnormalities: t(4;11)1
	Unfavourable 5/36	Philadelphia chromosom/bcr-abl translocation 1
Flt3 abnormalities	No Flt3 abnormality 17/36	
	Flt3-ITD 16/36	
	Asp835 mutation 3/36	

**Response to chemotherapy**	Patients receiving chemotherapy 23/39	Patients receiving chemotherapy 6/8
	Complete remission after first induction 9/23	Death due to primary resistance or leukemia relapse within 6 months 4/6

Totally 73 AML and 16 ALL images were included in the analysis. All ALL patients had B-cell disease and two patients comprised the bcr-abl fusion product. ^1^Patients were classified as positive when at least 20% of the AML cells expressed the membrane molecule [55]. ^2^Cytogenetic abnormalities in AML cells were classified according to Wheatley et al. [56].

## References

[B1] O'Farrell PH (1975). High Resolution two-dimensional electrophoresis of proteins. J Biol Chem.

[B2] Gorg A, Weiss W, Dunn M (2004). Current two-dimensional electrophoresis technology for proteomics. Proteomics.

[B3] Celis J, Moreira J, Cabezon T, Gromob P, Friis R, Rank F, Gromova I (2005). Identification of extracellular and intracellular signaling components of the mammary adipose tissue and its interstitial fluid in high risk breast cancer patients: toward dissecting the molecular circuitry of epithelial-adipocyte stromal cell interactions. Mol Cell Proteomics.

[B4] Boyd R, Adams P, Patel S, Loader J, Berry J, Redpath N, Poyser H, Fletcher G, Burgess N, Stamps A, Hudson L, Smith P, Griffiths M, Willis T, Karran E, DG O, Catovsky D, Terrett J, Dyer M (2003). Proteomic Analysis of the cell-surface membrane in chronic lymphocytic leukemia: identification of two novel proteins, BCNP1 and MIG2B. Leukemia.

[B5] Gjertsen BT, Oyan A, Marzolf B, Hovland R, Gausdal G, Doskeland SO, Dimitrov K, Golden A, Kalland K, Hood L, Bruserud Ø (2002). Analysis of acute myelogenous leukemia: preparation of samples for genomic and proteomic analysis. J Hematother Stem Cell Res.

[B6] Sjoholt G, Ånensen N, Wergeland L, McCormak E, Bruserud Ø, Gjertsen BT (2005). Proteomics in acute myelogenous leukemia (AML): methodological strategies and identification of protein targets for novel antileukemic therapy. Current Drug Targets.

[B7] Schmidt C, Przybylski G, Tietze A, Oettle H, Siegert W, Ludwig W (1996). Acute myeloid and T-cell acute lymphoblastic leukaemia with aberrant antigen expression exhibit similar TCRdelta gene rearrangements. Br J Haematol.

[B8] Hanash S (2003). Disease proteomics. Nature.

[B9] Conrads T, Zhou M, Petricoin E, Liotta L, Veenstra T (2003). Cancer diagnosis using proteomic patterns. Expert Rev Mol Diagn.

[B10] Cui J, Wang J, He K, Jin B, Wang H, Li W, Kang L, Hu M, Li H, Yu M, Shen B, Wang G, Zang X (2004). Proteomic analysis of human acute leukemia cells: insight into their classification. Clin Cancer Res.

[B11] Garell J (1979). Two-dimensional gel electrophoresis and computer analysis of proteins synthesized by clonal cell lines. J Biol Chem.

[B12] Curch S (2004). Advances in two-dimensional gel matching technology. Biochem Soc Trans.

[B13] Blose S, Hamburger S (1985). Computer-analyzed high resolution two-dimensional gel electrophoresis: a new window for protein research. Biotechniques.

[B14] Horgan G, Glasbey CA (1995). Uses of digital image analysis in electrophoresis. Electrophoresis.

[B15] Appel R, Hochstrasser D, Funk M, Vargas J, Pellegrini C, Muller A, Sherrer J (1991). The MELANIE Project: from a biopsy to automatic protein map interpretation by computer. Electrophoresis.

[B16] Anindya R, Kwan RL, Yarning H, Marten M, Babu R (2003). Analyzing Two-Dimensional Gel Images.

[B17] Schlags W, Walther M, Masree M, Kratzel M, Noe CR, Lachmann B (2005). Towards validating a method for two-dimensional electrophoresis/silver staining. Electrophoresis.

[B18] Grimwade D, Walker H, Oliver F, Wheatley K, Harrison C, Rees J, Hann I, Stevens R, Burnett A, Goldstone A (1998). The importance of diagnostic cytogenetics on outcome in AML: analysis of 1612 patients entered into the MRC AML 10 trial. The Medical Research Council Adult and Children's Leukaemia Working Parties; Blood.

[B19] Gilliland D, CT CJ, Felix C (2004). The Molecular Basis of Leukemia. Hematology (Am Soc Hematol Educ Program).

[B20] Chaladon Y, Schwaller J (2005). Targeting mutated protein tyrosine kinases and their signaling pathways in hematologic malignancies. Haematologica.

[B21] Lonning P (2004). Genes causing inherited cancer as beacons to identify the mechanisms of chemoresistance. Trends Mol Med.

[B22] Bode A, Dong Z (2004). Post-translational modifications of p53 in tumorigenesis. Nat Rev Cancer.

[B23] Harris S, Levine A (2005). The p53 pathway: positive and negative feedback loops. Oncogene.

[B24] Shen D, Real F, DeLeo A, Old L, Marks P, Rifkind R (1983). Protein p53 and inducer-mediated erythroleukemia cell commitment to terminal cell division. Proc Natl Aca Sci USA.

[B25] Rizzo M, Zepparoni A, Cristofanelli B, Scardigli R, Crescenzi M, Blandino G, Giuliacci S, Ferrari S, Soddu S, Sacchi A (1998). Wt-p53 action in human leukemia cell lines corresponding to different stages of differentiation. Br J Cancer.

[B26] Gonzalez RC, Woods RE (2002). Digital Image Processing.

[B27] Veterling WT, Flannery BP (2002). Numerical Recipes in C++.

[B28] Irish J, Hovland R, Krutzik P, Perez O, Bruserud Ø, Gjertsen B, Nolan G (2004). Single Cell profiling of potentiated phospho-protein networks in cancer cells. Cell.

[B29] Adjei A, Hidalgo M (2005). Intracellular signal transduction pathway proteins as targets for cancer therapy. J Clin Oncol.

[B30] Bourdon J, Fernandes K, Murray-Zmijewski F, Liu G, Diot A, Xirodimas D, Saville M, Lane D (2005). p53 isoforms can regulate p53 transcriptional activity. Genes Dev.

[B31] Meek D (1998). Multisite phosphorylation and the integration of stress signals at p53. Cell Signal.

[B32] Stone R, O'Donnell M, Sekeres M (2004). Acute myeloid leukemia. Hematology (Am Soc Hematol Educ Program).

[B33] Hoelzer D, Gokbuget N, Ottmann O, Pui C, Relling M, Appelbaum F, van Dongen J, Szczepanski T (2002). Acute lymphoblastic leukemia. Hematology (Am Soc Hematol Educ Program).

[B34] Waele MD, Renmans W, Gucht KV, Jochmans K, Schots R, Otten J, Trullemans F, Lacor P, Riet IV (2001). Growth factor receptor profile of CD34+ cells in AML and B-lineage ALL and in their normal bone marrow counterparts. Eur J Haematol.

[B35] Sakhinia E, Faranghpour M, Yin JL, Brady G, Hoyland J, Byers R (2005). Routine expression profiling of microarray gene signatures in acute leukaemia by real-time PCR of human bone marrow. Br J Haematol.

[B36] Bennett J, Catovsky D, Daniel M, Flandrin G, Galton D, Gralnick H, Sultan C (1991). Proposal for the recognition of minimally differentiated acute myeloid leukemia (AML-M0). Br J Haematol.

[B37] Bennett J, Catovsky D, Daniel M, Flandrin G, Galton D, Gralnick H, Sultan C (1976). Proposals for the classification of the acute leukaemias. French-American-British (FAB) co-operative group. Br J Haematol.

[B38] Bullinger L, Dohner K, Beir E, Frohling S, Schlenk R, Tibshirani R, Dohner H, Pollack J (2004). Use of gene-expression profiling to identify prognostic subclasses in adult acute myeloid leukemia. N Engl J Med.

[B39] Valk P, Verhaak R, Beijen M, Erpelinck C, van Waalwijk, van Doorn S, Khosrovani B, Boer J, Beverloo H, Moorhouse M, van der Spek P, Lowenberg B, Delwel R (2004). Prognostically useful gene-expression profiles in acute myeloid leukemia. N Engl J Med.

[B40] Soddu S, Blandino G, Citro G, Scardigli R, Piaggio G, Ferber A, Calabretta B, Sacchi A (1994). Wild-type p53 gene expression induces granulocytic differentiation of HL-60 cells. Blood.

[B41] Tang P, Wang F (2000). Induction of IW 32 erythroleukemia cell differentiation by p53 is dependent on protein tyrosine phosphatase. Leukemia.

[B42] Lin T, Chao C, Saito S, Mazur S, Murphy M, Apella E, Xu Y (2005). p53 induces differentiation of mouse embryonic stem cells by suppressing Nanog expression. Nat Cell Biol.

[B43] Harris N, Jaffe E, Diebold J, Flandrin G, Muller-Hermelink H, Vardiman J, Lister T, Bloomfield C (1997). 1999 World Health Organization Classification of Neoplastic Diseases of the Hematopietic and Lymphoid Tissues: report on the Clinical Advisory Committee Meeting, Arlie House, Virginia. J Clin Oncol.

[B44] Belle WV, Sjøholt G, Ånensen N, Høgda KA, Gjertsen BT (2006). Adaptive Contrast Enhancement of Two-Dimensional Electrophoretic Gels Facilitates Visualization, Orientation and Alignment.

[B45] Wang X, Feng DD (2005). Hybrid Registration for Two-Dimensional Gel Protein Images. Third Asia Pacific Bioinformatics Conference (APBC2005).

[B46] Big O Notation. http://en.wikipedia.org/wiki/Big_O_notation.

[B47] Knuth D (1997). The Art of Computer Programming.

[B48] Branco J, Croux C, Filzmoser P, Oliviera M (2005). Robust Canonical Correlations: A Comparative Study. Computational Statistics.

[B49] Dehon C, Filzmoser P, Croux C, Kiers HAL, Rasson JP, Groenen PJF, Schrader M (2000). Data Analysis, Classification, and Related Methods, chap Robust Methods for Canonical Correlation Analysis.

[B50] Stutz J, Cheeseman P (1994). Maximum Entropy and Bayesian Methods.

[B51] Cheeseman P, Stutz J (1996). Advances in Knowledge Discovery and Data Mining.

[B52] Hough P (1962). Methods and Means for Recognizing Complex Patterns. US Patent 3,069,654.

[B53] Conradsen K, Pedersen J (1992). Analysis of two-dimensional electrophoresis gels. Biometrics.

[B54] Kenny J, Keeping E (1962). The Standard Deviation and Calculation of the Standard Deviation.

[B55] Bene M, Catoldi G, Knapp W, Ludwig W, Matutes E, Orfao A, Veer MV (1995). Proposals for the immunological classification of acute leukemias. European Group for the Immunological Characterization of Leukemias (EGIL). Leukemia.

[B56] Wheatley K, Burnett A, Goldstone A, Gray R, Hann I, Harrison C, Rees J, Stevens R, Walker H (1999). A simple, robust, validated and highly predictive index for the determination of risk-directed therapy in acute myeloid leukaemia derived from the MRC AML 10 trial. United Kingdom Medical Research Council's Adult and Childhood Leukaemia Working Parties. Br J Haematol.

[B57] Øystein Bruserud, Hovland R, Wergeland L, Huang T, Gjertsen BT (2003). FItS-mediated signaling in human acute myelogenous leukemia (AML) blasts: a functional characterization of Flt3-ligand effects in AML cell populations with and without genetic Flt3 abnormalities. Haematologica.

[B58] Ersvaer E, Bertelsen L, Espenes L, Bredholt T, Boe S, Iversen B, Øystein Bruserud, Ulvestad E, Gjertsen BT (2004). Characterization of ribosomal P autoantibodies in relation to cell destruction and autoimmune disease. Scan J Immunol.

[B59] Gjertsen BT, Mellgren G, Otten A, Maronde E, Genieser HG, Jastroff B, Vintermyr O, McKnight GS, Doskeland SO (1995). Novel (Rp)-cAMPS analogs as tools for inhibition of cAMP-kinase in cell culture. Basal cAMP-kinase activity modulates interleukin-1 *β *action. J Biol Chem.

[B60] Research Systems Inc (RSI) C Boulder IDL, The Interactive Data Language, v6.1.

[B61] Blender 3D. http://www.blender3d.org/.

[B62] Mplayer Headquarters. http://www.mplayerhq.hu/.

[B63] Winzip – The ZIP Utility for windows. http://www.winzip.com/.

[B64] ZipIt: Macintosh Compression Utility. http://www.maczipit.com/.

