# Supplementary figures and images for: Correlation analysis of two-dimensional gel electrophoretic protein patterns and biological variables
Source: BMC Bioinformatics. 2006 Apr 10;7:198. doi: 10.1186/1471-2105-7-198 (PMC1559651; doi:10.1186/1471-2105-7-198)

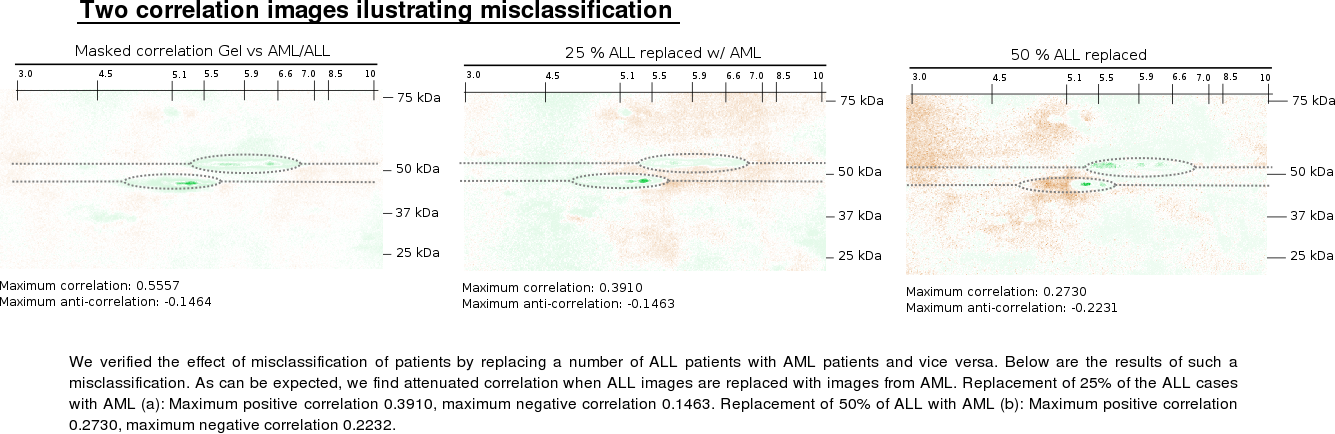

Supplement: Additional file 3 — Correlation images illustrating impact of misclassification. The impact of wrong ALL versus AML diagnosis was examined by random swapping ALL and AML labels in the AML/ALL versus 2DE image correlations. This results in lower correlation values as expected. [file 1471-2105-7-198-S3.png]

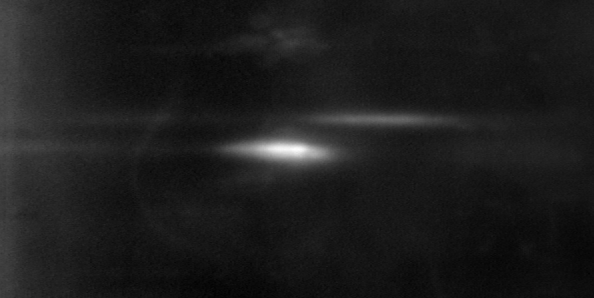

Supplement: Additional file 2 — Spot shapes. Two images showing the difference in spot sizes between M0/M1/M2 and M4/M5 samples. The process of the changing spot distribution can be visualized by sorting all images according to their FAB classification and then showing them chronologically. This is visualized in a small movie. The two images and the movie are contained within a zip file. It can be extracted using unzip [63,64]. The movie can be played with mplayer [62]. [file 1471-2105-7-198-S2.zip › class0.png]

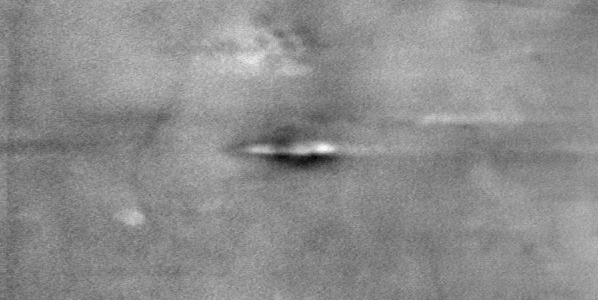

Supplement: Additional file 2 — Spot shapes. Two images showing the difference in spot sizes between M0/M1/M2 and M4/M5 samples. The process of the changing spot distribution can be visualized by sorting all images according to their FAB classification and then showing them chronologically. This is visualized in a small movie. The two images and the movie are contained within a zip file. It can be extracted using unzip [63,64]. The movie can be played with mplayer [62]. [file 1471-2105-7-198-S2.zip › class1-class0.png]

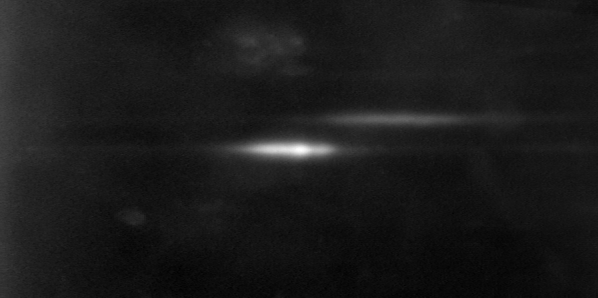

Supplement: Additional file 2 — Spot shapes. Two images showing the difference in spot sizes between M0/M1/M2 and M4/M5 samples. The process of the changing spot distribution can be visualized by sorting all images according to their FAB classification and then showing them chronologically. This is visualized in a small movie. The two images and the movie are contained within a zip file. It can be extracted using unzip [63,64]. The movie can be played with mplayer [62]. [file 1471-2105-7-198-S2.zip › class1.png]

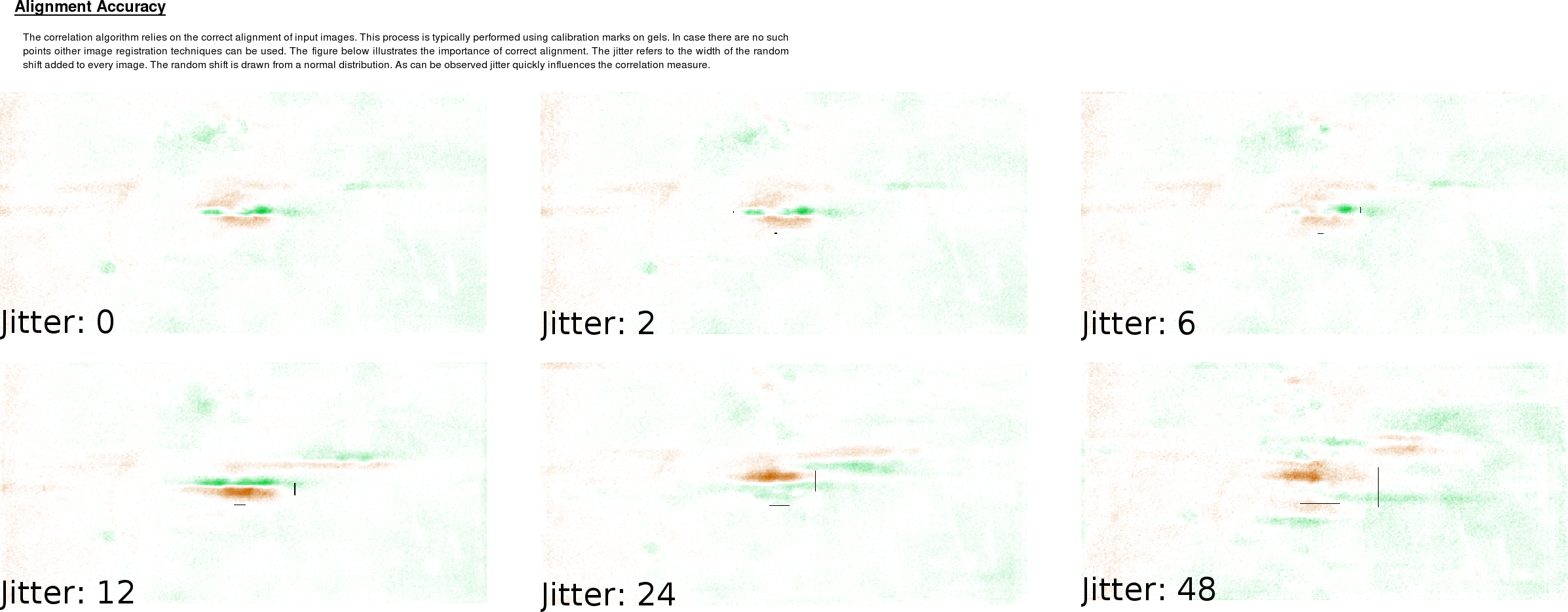

Supplement: Additional file 4 — Alignment accuracy. The correlation algorithm relies on the correct alignment of input images. This process is typically performed using calibration marks on gels. The additional data illustrates the importance of correct alignment. [file 1471-2105-7-198-S4.png]
