# Supplementary material for: Correlation analysis of two-dimensional gel electrophoretic protein patterns and biological variables
Source: BMC Bioinformatics. 2006 Apr 10;7:198. doi: 10.1186/1471-2105-7-198 (PMC1559651; doi:10.1186/1471-2105-7-198)
Supplement: Additional file 2 — Spot shapes. Two images showing the difference in spot sizes between M0/M1/M2 and M4/M5 samples. The process of the changing spot distribution can be visualized by sorting all images according to their FAB classification and then showing them chronologically. This is visualized in a small movie. The two images and the movie are contained within a zip file. It can be extracted using unzip [63,64]. The movie can be played with mplayer [62]. [file 1471-2105-7-198-S2.zip › index.html]

# Correlation Analysis of Two-Dimensional Gel Electropheretic Protein Patterns and Biological Variables: Additional Material

## 2. Two correlation images of mean  spot shape

We 
created the mean gel image of all gels annotated M0,
M1 or M2 (left) as well as those annotated M4 or M5 (right).

|  |  |
| --- | --- |
|  |  |

The difference between the two images is visualized below

The
process of changing spot distribution can be visualized by sorting all
images according to their fab classification and showing them
chroinologically. This is visualized in the next small movie.

  
