# Supplementary material for: Correlation analysis of two-dimensional gel electrophoretic protein patterns and biological variables
Source: BMC Bioinformatics. 2006 Apr 10;7:198. doi: 10.1186/1471-2105-7-198 (PMC1559651; doi:10.1186/1471-2105-7-198)
Supplement: Additional file 5 — Source material. The source material includes correlation analysis, image coloring, Gaussian bumps and the simulated images. The source is contained within a .html file. The algorithm is implemented in IDLv6.1 [60]. [file 1471-2105-7-198-S5.html]

## Source Material

### 5.1 The analysis method

The
algorithm is implemented in IDLv6.1 [IDL] and takes two arguments. The
first argument is the gel-stack, which is a three dimensional space.
First dimension is the gel number, the second and third dimensions are
the x and y-axis of the gels. The second argument is a vector
describing the result of the different gels. The presented algorithm
makes use of a mean scaling.

PRO
correlate\_images, all, result  
  d =
size(all,/dim)  
  VX =
d[1]  
  VY =
d[2]  
  ;
normalize the background  
  for i
= 0, d[0] - 1 do begin  
   
all[i,\*,\*] /= mean(all[i,\*,\*])  
  endfor  
  ; Rho
correlation  
 
cor\_pic = make\_array(VX,VY,/double,value=0.0)  
  f\_pic
= make\_array(VX,VY,value=0.0)  
  for x
= 0, VX - 1 do begin  
   
for y = 0, VY - 1 do begin  
     
r = r\_correlate(reform(all[\*,x,y]),result)  
     
cor\_pic[x,y]=r[0]  
     
f\_pic[x,y]=1.0-r[1]  
   
endfor  
  endfor  
  ; we
are interested in correlations with high variance on gel  
 
var\_pic = make\_array(VX,VY,/double,value=0.0)  
  for x
= 0, VX - 1 do begin  
   
for y = 0, VY - 1 do begin  
     
var\_pic[x,y]=stddev(all[\*,x,y])  
   
endfor  
  endfor  
 
var\_pic <= 1.0  
  f\_pic
\*= var\_pic  
 
cor\_pic <= 1.0  
 
cor\_pic >= -1.0  
 
show\_correlation, cor\_pic, f\_pic  
end

### 5.2. Gauss Bumps

function gauss2d,
sx, sy, cx, cy, wx, wy, a  
  im =
float(make\_array(sx,sy,value=0.0))  
  for x
= 0, sx - 1 do begin  
   
for y = 0, sy - 1 do begin  
     
im[x,y]=float(((cx-x)/wx)^2 + ((cy-y)/wy)^2)  
   
endfor  
  endfor  
  im =
-im/2  
  im =
exp(im)  
  im \*= a  
 
return, im  
end

### 5.3. Simulated Gel Stack

function
create\_set, nr, sx, sy, wx1, wx2, wy1, wy2, a1, a2  
  all = make\_array(nr,sx,sy,value=0.0)  
  for i = 0, nr - 1 do begin  
    wx = wx1 + i\*(wx2-wx1)/nr  
    wy = wy1 + i\*(wy2-wy1)/nr  
    a = a1 + i\*(a2-a1)/nr  
    all[i,\*,\*] = gauss2d(sx, sy, sx/2, sy/2, wx, wy, a)  
  endfor  
  return, all  
end  
  
set1 = create\_set(15, 600.0, 400.0, 10.0, 100.0, 10.0, 100.0, 5.0, 1.0)  
set2 = create\_set(15, 300.0, 300.0, 10.0, 40.0, 40.0, 10.0, 5.0, 5.0)  
set3 = create\_set(15, 300.0, 300.0, 20.0, 20.0, 20.0, 20.0, 5.0, 5.0)  
set4 = create\_set(15, 300.0, 300.0, 20.0, 20.0, 20.0, 20.0, 1.0, 1.0)  
  
set1[\*,0:299,0:299] += set2[\*,\*,\*]  
set1[\*,300:599,0:299] += set3[\*,\*,\*]  
set1[\*,300:599,100:399] += set4[\*,\*,\*]  
for i = 0, 14 do begin  
  set1[i,0+i\*10:299+i\*10,200:399]+=set3[i,\*,50:249]  
endfor  
for i = 0, 14 do begin  
  set1[i,\*,\*]/=double(i)  
endfor  
set1 = relative(set1)  
result1 = findgen(15)  
correlate\_images, set1, result1  
end

### 5.4. Creation of green/brown images

PRO
show\_correlation, cp, t\_pic  
 
cor\_pic = cp  
  DDD =
size(cp,/dim)  
  VX =
ddd[0]  
  VY =
ddd[1]  
  ; the
normal one  
  shown
= make\_array(3,VX,VY,/double,value=255.0)  
  multi
= 1.0 / max(abs(cor\_pic))  
  multi
= 1.0 / max(abs(cor\_pic))  
 
shown[0,\*,\*] += (cor\_pic[\*,\*] < 0) \* multi \* 55  
 
shown[1,\*,\*] += (cor\_pic[\*,\*] < 0) \* multi \* 155  
 
shown[2,\*,\*] += (cor\_pic[\*,\*] < 0) \* multi \* 255  
 
shown[0,\*,\*] -= (cor\_pic[\*,\*] > 0) \* multi \* 255  
 
shown[1,\*,\*] -= (cor\_pic[\*,\*] > 0) \* multi \* 55  
 
shown[2,\*,\*] -= (cor\_pic[\*,\*] > 0) \* multi \* 205  
 
window, 1, title='Correlation', ret=2, xsize=vx, ysize=vy  
  shown
>= 0  
  shown
<= 255  
  tvscl,
shown, /true  
  ; only
the significant correlation  
 
wcor\_pic = double(cor\_pic) \* double(t\_pic)  
  shown
= make\_array(3,VX,VY,/double,value=255.0)  
  multi
= 255.0 / max(abs(wcor\_pic))  
 
shown[0,\*,\*] += (wcor\_pic[\*,\*] < 0) \* multi \* 55  
 
shown[1,\*,\*] += (wcor\_pic[\*,\*] < 0) \* multi \* 155  
 
shown[2,\*,\*] += (wcor\_pic[\*,\*] < 0) \* multi \* 255  
 
shown[0,\*,\*] -= (wcor\_pic[\*,\*] > 0) \* multi \* 255  
 
shown[1,\*,\*] -= (wcor\_pic[\*,\*] > 0) \* multi \* 55  
 
shown[2,\*,\*] -= (wcor\_pic[\*,\*] > 0) \* multi \* 205  
 
window, 3, title='Significant Correlations', ret=2, xsize=vx, ysize=vy  
  tvscl,
shown, /true  
  shown
= bytscl(shown)  
 
profiles, cor\_pic  
  ;
significance  
 
window, 2, title='Significance', ret=2, xsize=vx, ysize=vy  
  tvscl,
t\_pic  
 
profiles, t\_pic  
end

  

### References

IDL - Research
Systems Inc (RSI) C Boulder: IDL, The Interactive Data Language, v6.1.  
  
